# Programmable Compliance in Small‐Diameter Vascular Grafts by Design of Melt‐Electrowritten Scaffold Architectures for In Situ Tissue Engineering

**DOI:** 10.1002/adhm.202502038

**Published:** 2025-09-12

**Authors:** Kilian Maria Arthur Mueller, Christina Ahrens, Linda Grefen, Salma Mansi, Dario Arcuti, Elena De‐Juan‐Pardo, Felix Kur, Christian Hagl, Petra Mela

**Affiliations:** ^1^ Chair of Medical Materials and Implants Department of Mechanical Engineering TUM School of Engineering and Design Munich Institute of Biomedical Engineering (MIBE) Munich Institute of Integrated Materials Energy and Process Engineering (MEP) Technical University of Munich 85748 Garching Germany; ^2^ Department of Cardiac Surgery LMU University Hospital 81377 Munich Germany; ^3^ DZHK (German Centre for Cardiovascular Research) Partner Site Munich Heart Alliance 80802 Munich Germany; ^4^ T3mPLATE Harry Perkins Institute of Medical Research Queen Elizabeth II Medical Centre and University of Western Australia Centre for Medical Research The University of Western Australia Perth WA 6009 Australia; ^5^ School of Engineering The University of Western Australia Perth WA 6009 Australia; ^6^ Curtin Medical School Curtin University Perth WA 6102 Australia

**Keywords:** access graft, biofabrication, compliance, melt electrowriting, vascular graft

## Abstract

In clinical practice, synthetic vascular grafts are advantageous due to their immediate availability but are burdened by high failure rates in small‐diameter settings because of thrombogenicity, infections, and intimal hyperplasia (IH). A mismatch in compliance between graft and host vessel has been identified as a major contributor to the development of IH. Here, we propose a design strategy to fabricate polymeric small‐diameter vascular graft scaffolds with programmable compliance based on a helical microfiber architecture via melt electrowriting (MEW). By controlling the fiber winding angle, this design strategy exploits, for the first time, the mechanical structure‐function relationship of MEW scaffolds to enable tailored compliance covering the physiological range of arteries and veins. This concept is complemented by an integrated microporous MEW graft wall, potentially enabling in situ tissue engineering to combine the advantages of synthetic (off‐the‐shelf) and autologous (living) grafts. Leveraging this, a gradient is introduced in the fiber architecture to achieve arteriovenous grafts matching the compliance of the target vessels at their ends (arterial vs. venous compliance) with a continuous smooth transitional region in between. The potential for clinical translation is demonstrated in vitro by assessing suture‐retention strength, anti‐kinking properties, burst pressure, and cannulation behavior.

## Graphical abstract

Small‐diameter vascular grafts with compliance tunable by design are fabricated via melt electrowriting. By controlling the winding angle of intertwined helical fibers, grafts with compliances matching those of human vessels, from veins to arteries, are realized. This holds the potential of avoiding a compliance mismatch, which has been identified as a major driver for graft failure.

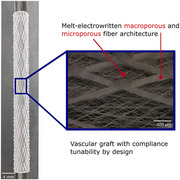

## 1 Introduction

Autologous arteries or veins are the current gold standard for small‐diameter vessel substitutes, e.g., for coronary artery bypass grafting (CABG).^[^ ^1^ ^]^ However, autografts are of limited availability, may be of unsuitable quality, and can cause donor site morbidity.^[^ ^2^, ^3^ ^]^ Therefore, synthetic grafts evolved as a promising off‐the‐shelf alternative with immediate availability. Despite their satisfactory performance in large‐diameter settings, synthetic grafts are plagued by low patency rates when used as small‐diameter prostheses, mainly caused by surface thrombogenicity and the development of intimal hyperplasia (IH).^[^ ^4^ ^]^

Tissue engineering (TE) holds the potential of providing artificially produced living vessels to the patients.^[^ ^5^ ^]^ Specifically, the paradigm of in situ TE synergizes the immediate availability of synthetic grafts with the long‐term functionality of living autologous vessels.^[^ ^6^ ^]^ In situ tissue‐engineered grafts are microporous, initially cell‐free scaffolds, and once implanted, they exploit the host's innate regenerative potential to stimulate endogenous tissue formation while degrading.^[^ ^7^, ^8^ ^]^ Careful scaffold design is imperative, both from a biological and a mechanical perspective. In fact, a mismatch in compliance, that is, the radial extensibility of a vessel in response to a luminal pressure wave, has been identified as a major contributing factor to IH.^[^ ^9^, ^10^, ^11^ ^]^

Indeed, experimental studies showed that small‐diameter tissue‐engineered vascular grafts (TEVGs) with the same compliance resulted in viable neotissue formation in the venous circulation, while they failed catastrophically when implanted in the arterial circulation of mice.^[^ ^12^ ^]^ Similarly, identical grafts had higher occlusion rates when implanted in the carotid than in the abdominal aorta of rats.^[^ ^13^ ^]^ The effect of compliance on the in vivo remodeling has been shown in a recent study, where compliance‐matched grafts resulted in more favorable remodeling compared to hypocompliant grafts.^[^ ^14^ ^]^ These findings highlight the pressing need for small‐diameter vascular grafts (SDVGs) whose compliance can be specifically tailored to the target vessel. This can also be extrapolated to larger diameters, such as the human ascending aorta, where comparatively stiff Dacron grafts are often used, resulting in up‐ and downstream cardiovascular problems as reported in the clinical literature.^[^ ^15^ ^]^

MEW is a powerful biofabrication technique that offers unique control over scaffold mechanics due to its precise microfiber placement.^[^ ^16^, ^17^ ^]^ Assisted by an electric field, MEW stacks polymer microfibers along predefined pathways into multi‐layered architectures, resulting in accurately defined macroporous scaffolds.^[^ ^16^, ^17^ ^]^ This has been extensively exploited for cardiovascular TE,^[^ ^18^ ^]^ e.g., for heart valves^[^ ^19^, ^20^, ^21^, ^22^ ^]^ and cardiac patches.^[^ ^23^, ^24^, ^25^, ^26^, ^27^, ^28^ ^]^

MEW is also gaining increasing interest for TE of SDVGs.^[^ ^29^, ^30^, ^31^, ^32^, ^33^, ^34^, ^35^, ^36^, ^37^, ^38^, ^39^ ^]^ Differently from other properties such as suture retention strength and burst strength, the compliance has not been consistently considered in the MEW scaffold's design phase, with only a minority of these studies addressing this property,^[^ ^34^, ^39^ ^]^ despite its importance for the functionality of the graft. The same holds for anti‐kinking behavior,^[^ ^39^ ^]^ a critical property for clinical translation, considering that the first clinical application of a TEVG would be as a dialysis shunt. Surprisingly, and in strong contrast to the widely emphasized potential of MEW to fabricate scaffolds with a tunable mechanical structure‐property relationship, no MEW pattern has yet been designed to obtain a programmable compliance across the physiological range, encompassing arterial and venous values, nor to confer anti‐kinking properties to the grafts. The unique potential of MEW for mechanically superior SDVG scaffolds remains, therefore, largely untapped. For example, Federici et al., created vascular grafts with different compliances, however, by varying the thickness of an abluminal electrospun layer covering a MEW construct^[^ ^39^ ^]^ similar to what Colino et al. proposed,^[^ ^40^ ^]^ in that case with a luminal warp‐knitted mesh instead of the MEW construct. The electrospun layer also served the function of covering the macroporous MEW component to enable the in situ TE approach. On the contrary, Weekes et al. chose to follow the in vitro TE approach,^[^ ^34^ ^]^ where the macroporous MEW scaffold would be embedded in neo‐tissue produced in a bioreactor. Their graft designs consisted either of a boxed pattern or of a sinusoidal circumferential pattern with straight perpendicular (longitudinal) fibers. Although they investigated the compliance resulting from two different fiber densities in both of the configurations, the in vitro cultivation to obtain grafts with tissue was not performed, and, therefore, the compliance of the final graft as to be implanted is not known. In vivo studies involving MEW scaffolds only report on compliance at the site of implantation or after explantation^[^ ^33^, ^37^ ^]^ however, without further elucidating to which extent the MEW scaffold designs contributed to the grafts’ compliance.

Here, we propose an advanced scaffold strategy for the fabrication of tunable SDVGs with the capability to match the compliance of the native target vessel only by design of the MEW fiber architecture, over the entire anatomical range (venous to arterial compliance). We comply with the requirements of in situ TE not by adding an electrospun component for microporosity, but instead by providing an integrated microporous MEW layer in the scaffolds. This is the first design strategy that truly exploits the programmable structure‐property relationship of a purely melt‐electrowritten vascular graft scaffold. The resulting grafts are thoroughly characterized in agreement with ISO 7198 and show excellent handling and anti‐kinking behavior. Ultimately, the grafts’ design strategy offers the option of introducing a compliance gradient, e.g., for arteriovenous grafts (AVGs) where the implant is connecting an artery and a vein. Considering the translational routine of validating novel graft concepts first as arteriovenous grafts, with their lower risk profile as e.g., compared to coronary bypass grafts, we ultimately explore a graft design strategy that offers the option of introducing a compliance gradient to respect the different compliance at the anastomoses i.e., that of an artery versus that of a vein.

## 2 Results and Discussion

### 2.1 Fiber Winding Angle Tunes Compliance Characteristics

**Figure 1:**
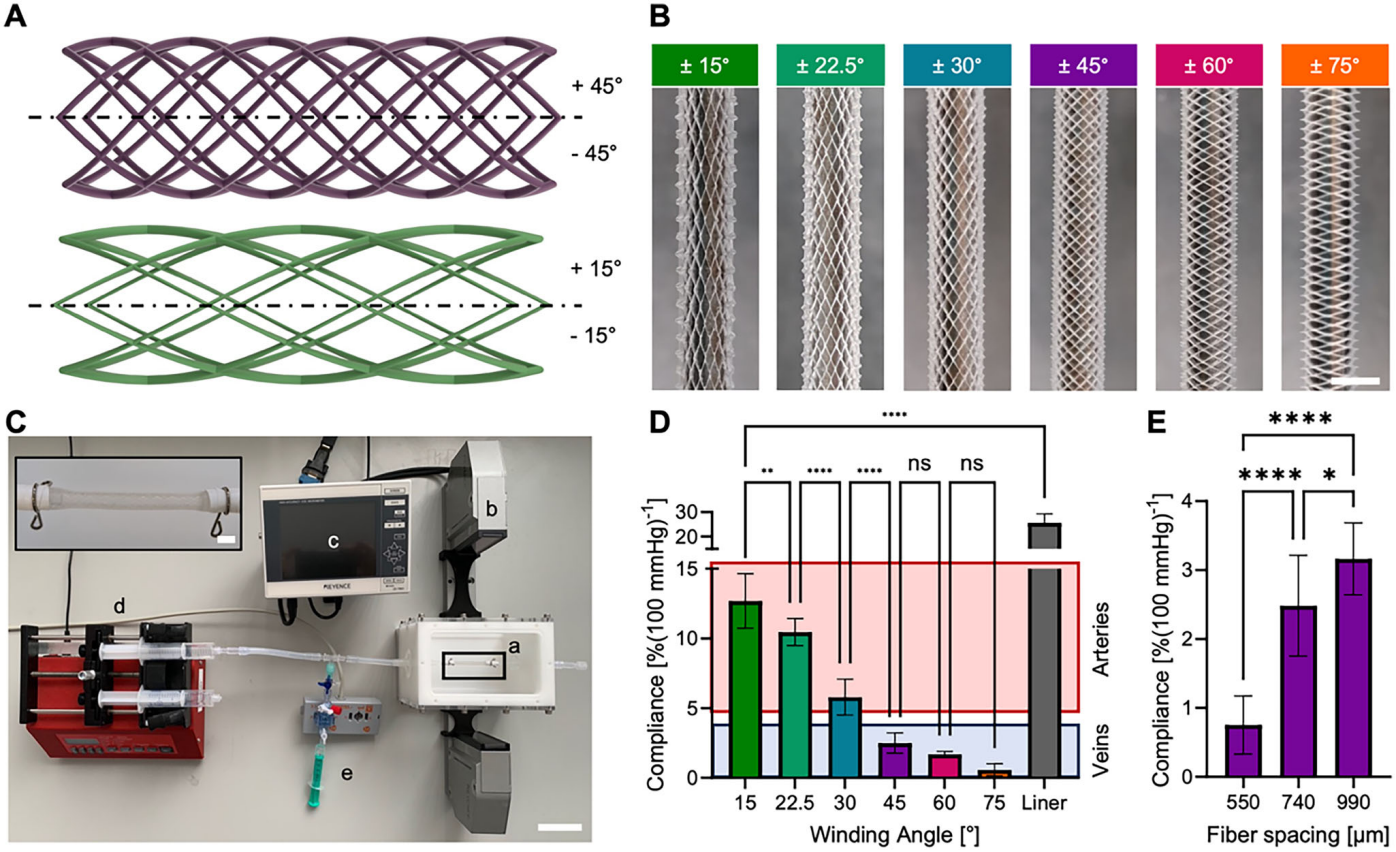
Fiber winding angle controls the compliance of melt‐electrowritten tubular scaffolds. A) Schematic representation of tubular architectures with two complementary fiber winding angles (purple: ±45°, green: ±15°, fiber diameters not to scale). B) Macroporous MEW scaffolds with fiber winding angles ranging from ±15° to ±75° on 4 mm diameter collectors (scale bar 4 mm). C) The compliance was determined by mounting a) the scaffolds orthogonal to the light path of b) an optical micrometer. c) A control unit visualized the changes in scaffold diameter in response to varying luminal pressures applied via d) a programmable syringe pump and recorded via e) a pressure sensor (scale bar 5 cm, inset scale bar 4 mm). D) Compliance obtained with different fiber angles covered the range reported for human arteries (red shaded box) and veins (blue shaded box). E) Varying the interfiber distance while keeping the winding angle constant (here ±45°) is an additional option for fine‐tuning the compliance.

We hypothesized that controlling the winding angle of linear melt‐electrowritten fibers along the graft's longitudinal axis allows to tune the compliance. Building on our previously reported software platform for MEW constructs,^[^ ^35^ ^]^ we generated fiber architectures for tubular scaffolds from multiple intertwined helices with fiber winding angles ranging from ±15° to ±75° (**Figure** **1**A,B). These linear fiber paths were connected with circular segments at the ends of the grafts to result in a single uninterrupted toolpath as required by the continuous fiber deposition nature of MEW. The distance between parallel fibers was set to 750 ± 50 µm. This quasi‐equidistant setting was dictated by the geometric restriction of keeping the graft's circumference constant for the different winding angles while ensuring a homogenous fiber density across the grafts. These programmed fiber architectures were fabricated by precisely depositing polycaprolactone (PCL) microfibers onto 4 mm diameter cylindrical collectors. 15 layers of fibers with a diameter of 24.2 ± 1.6 µm were stacked. The developed parametric software platform can generate G‐codes for grafts with any desired length and diameter, provided that an adequate mandrel is used.

In accordance with ISO 7198, the compliance of the constructs was determined by measuring the change in diameter in response to cyclic changes in the luminal pressure from 80 to 120 mmHg created by inflating a high‐compliance silicone liner (25.7 ± 3.5%(100 mmHg)^−1^) inside the scaffold (Figure 1C). Here, we found compliances ranging from 12.7 ± 2.0%(100 mmHg)^−1^ for the ± 15° scaffolds down to 0.6 ± 0.4%(100 mmHg)^−1^ for scaffolds with ± 75° fiber winding angles (Figure 1D). This tunable structure‐property relationship allows to cover the range of compliances of native human arteries (4.7–17.0%(100 mmHg)^−1^)^[^ ^41^, ^42^, ^43^, ^44^, ^45^, ^46^, ^47^, ^48^, ^49^ ^]^ and veins (0.7–3.7%(100 mmHg)^−1^)^[^ ^47^, ^48^, ^49^, ^50^, ^51^ ^]^ in contrast to the stiff clinically used synthetic grafts made of polytetrafluoroethylene (PTFE) (0.2–0.9%(100 mmHg)^−1^) or Dacron (0.8–1.9%(100 mmHg)^−1^).^[^ ^48^, ^49^, ^52^ ^]^ Testing the grafts at lower pressure conditions (50/90 mmHg) and higher pressure conditions (110/150 mmHg), as suggested in ISO 7198, showed a slight trend toward increasing compliance for scaffolds with low fiber winding angles (15° and 22.5°) for an increase in mean pressure while higher fiber angles (30°, 45°, 60°, 75°) presented a minor trend toward decreasing compliance (Figure S1, Supporting Information).

Controlling the winding angle of linear fibers, as shown here, is a simple yet effective design strategy to tune the compliance of macroporous scaffolds. In response to a luminal pressure pulse, the fibers of the graft wall reconfigure toward a more circumferential orientation to increase the graft's diameter. In this deformation mechanism, fibers with a larger winding angle and, therefore, with a bigger circumferential component, have less potential to reconfigure; hence, these grafts show reduced compliance. Conversely, with smaller fiber winding angles, higher compliance can be realized.

Additionally, the fiber spacing, i.e., the fiber density, can be used to fine‐tune the compliance, with larger fiber spacings resulting in higher compliances as exemplarily shown for a ±45° fiber winding angle (Figure 1E). This trend is in contrast to the work of Weekes et al., who showed a higher compliance for sinusoidal fiber patterns when the fiber spacing was decreased from 500 to 250 µm.^[^ ^34^ ^]^ The compliance could potentially be further adjusted by varying other MEW process parameters, such as the fiber diameter or the layer number. While this opens a large design space, MEW's inherent design limitations need to be considered, such as fiber bridging at a small interfiber distance.^[^ ^53^, ^54^ ^]^ On the other hand, too large fiber spacing might compromise the mechanical properties of the vascular graft, such as suture retention. In the following experiments, we proceeded with a fiber spacing of 750 ± 50 µm as this conveniently allowed to obtain compliance values in the physiologically relevant range.

### 2.2 Combining Macro‐ and Microporosity Forms Multiscale Scaffolds

**Figure 2:**
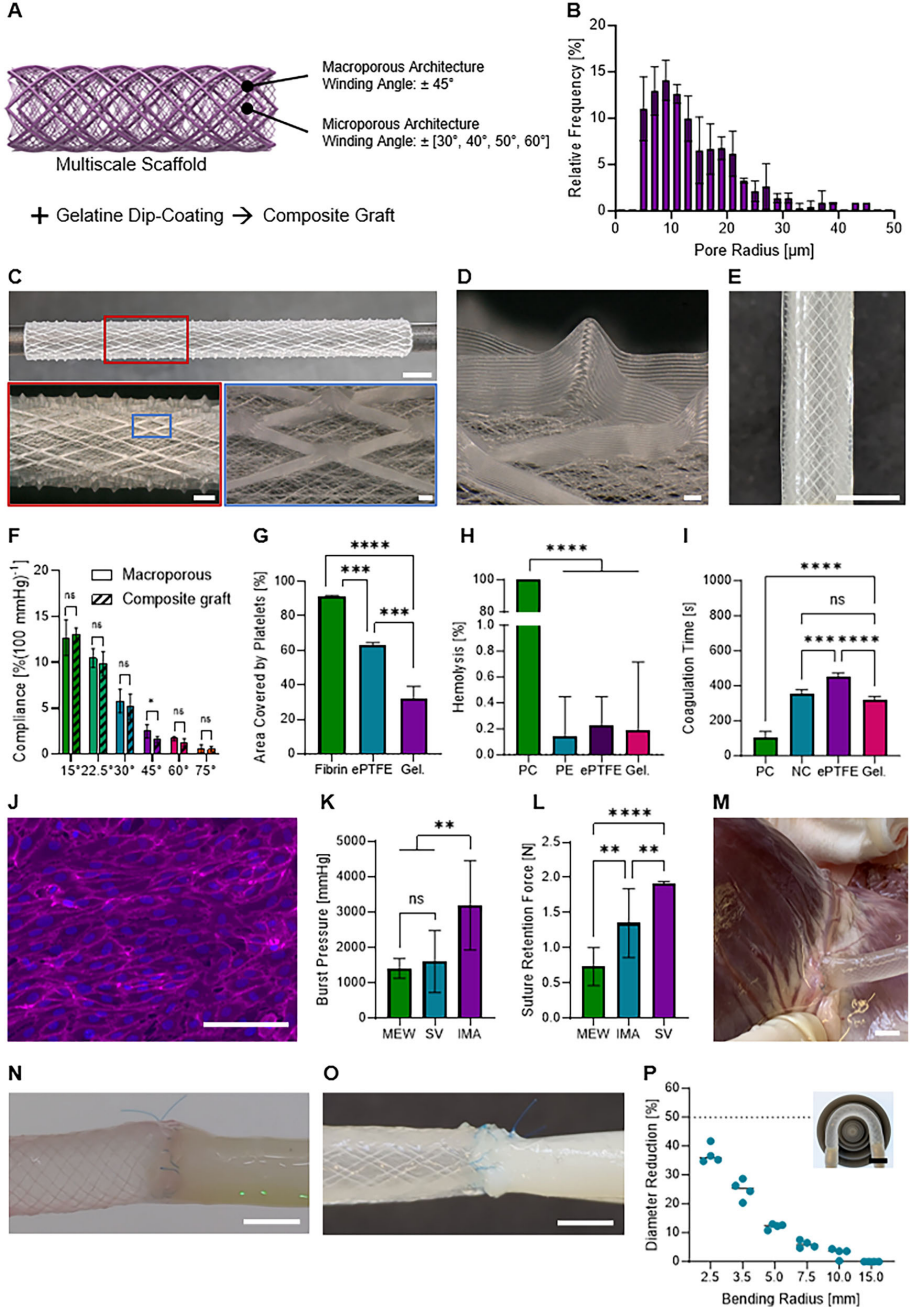
Compliant tubular scaffolds via MEW. A) Schematic representation of the design concept: combining an inner microporous MEW matrix with an outer macroporous MEW fiber architecture resulted in multiscale scaffolds that were dip‐coated in gelatine. B) Pore size distribution of the microporous matrix. C) MEW scaffold on a 4 mm diameter mandrel, highlighting accurate fiber stacking of the macroporous architecture (scale bars 4 mm, 1 mm, 100 µm). D) Magnified view of a fiber stack of the macroporous architecture (scale bar 100 µm). E) Scaffold after dip‐coating in gelatine (scale bar 4 mm). F) The composite scaffolds with a matching microporous fiber pattern showed compliances similar to those obtained for the macroporous scaffolds alone. G) Platelet adhesion on gelatine was lower than for the ePTFE and fibrin reference materials. H) The hemolysis induced by the gelatine matrix (Gel.) was comparable to polyethylene (PE) and expanded polytetrafluoroethylene (ePTFE) (positive control (PC): whole blood diluted in water). I) The coagulation time for contact with gelatine was found to be significantly longer than for the PC (fibrin) and similar to the negative control (NC: citrated platelet poor plasma) and ePTFE. J) The luminal surface of the graft supported endothelial layer formation in dynamic conditions (scale bar 100 µm). K) Burst pressures were comparable to those of the human saphenous vein (SV) but lower than those of the human internal mammary artery (IMA). Values for SV and IMA taken from Konig et al.^[^ ^46^ ^]^ Normal distribution was assumed for the data taken from the literature to perform statistical analysis. L) Suture retention force was lower than that of human IMA and SV. Values for SV taken from L'Heureux et al.^[^ ^50^ ^]^ and for IMA taken from Konig et al.^[^ ^46^ ^]^ Normal distribution was assumed for the data taken from the literature to perform statistical analysis. M) However, anastomosing to a porcine right coronary artery was unproblematic. The coronary artery was successfully pressurized via the graft (scale bar 4 mm). The grafts were also sutured to N) a human saphenous vein and O) a porcine femoral artery (scale bars 4 mm). P) No diameter reductions of more than 50% were observed, even for bending radii as low as 2.5 mm, indicating anti‐kinking behavior. The inset is showing bending of a graft around a 7.5 mm radius template (scale bar 10 mm).

Scaffolds for in situ TE require a microporosity to host cells for endogenous tissue formation.^[^ ^8^ ^]^ Therefore, we combined the macroporous fiber pattern with a microporous matrix, which was melt‐electrowritten in a first step to later form the graft's luminal side, following our approach to introduce microporosity for cell infiltration (**Figure** **2**A).^[^ ^35^ ^]^ To this end, we varied the MEW process parameters to obtain much smaller fibers with 9.2 ± 0.6 µm diameter and printed a fiber pattern based on the superposition of angularly shifted sets of parallel fibers (200 µm interfiber distance) on 4 mm diameter collectors. These parallel fiber sets were shifted ±5° and ±15° relative to the main fiber winding angle of the macroporous fiber pattern (e.g. ±[30°, 40°, 50°, 60°] for a ±45° macroporous pattern) to obtain a fiber pattern with an average fiber direction that matches the main fiber winding angle of the macroscopic fiber architecture. Five stacks of the microporous pattern resulted in a luminal layer with a thickness of 67 ± 8 µm and a pore size distribution that peaks at pore radii of 8 to 10 µm (14% relative frequency) with pores with radii between 4 and 15 µm contributing to 61% of the total number of pores (Figure 2B). This pore size will not change significantly if the main fiber winding angle is varied, but instead can be steered by changing the fiber spacing or the number of fiber sets.^[^ ^35^ ^]^

In a second step, we deposited the macroporous fiber pattern (as described in the section above) onto the microporous base matrix (Figure 2C,D). This is the first graft fabrication strategy building only on MEW design parameters to generate a microporous vessel wall with tunable compliance. An additional benefit of such a strategy is that no solvents were used, as in contrast to previous studies, where MEW has been combined with solution electrospinning to provide microporosity and tune the grafts’ compliance.^[^ ^30^, ^31^, ^39^ ^]^ Also, this work presents a fabrication workflow where the graft is produced in a single MEW print job, instead of joining two separately produced tubular MEW components and relying on friction in between them, as presented by Chen et al.^[^ ^37^ ^]^

Potentially, the MEW graft could be used as is, relying on hemostasis to seal the vessel wall, as analogously shown for electrospun vessel substitutes.^[^ ^55^, ^56^ ^]^ Alternatively, it can be complemented with a soft matrix, as long as this is not interfering with the compliance given by the MEW architecture. Here, we chose to embed the MEW scaffolds in gelatine by dip‐coating, which resulted in a total wall thickness of 1300 ± 100 µm (Figure 2E). The potential of gelatine for tissue‐engineered vascular grafts has been shown in literature. Gelatine encourages both the formation of the endothelium as well as infiltration and remodeling by smooth muscle cells.^[^ ^57^, ^58^ ^]^

For all fiber angles, the compliance of the composite grafts was well in agreement with that obtained for the macroporous architectures alone. Because the fiber pattern for the microporous matrix is based on the same design principle as in the macroporous architecture, and a soft gelatine hydrogel was used, the deformation mechanism of the macroporous patterns was not suppressed (Figure 2F). This mechanism was maintained after 1.2 million cycles of dynamic loading with a pressure profile of 80 to 120 mmHg at 2 Hz (Figure S2, Supporting Information) in contrast to commercially available grafts, which show loss of compliance after a few hours of pulsatile stimulation.^[^ ^59^ ^]^ However, when an isotropic microporous matrix (e.g. fiber angles: ±[11.25°, 33.75°, 56.25°, 78.75°]) was used, i.e. the microporous matrix was not matching the main fiber orientation given by the macroporous architecture, this drastically reduced the compliance of the composite graft (Figure S3, Supporting Information). This is because the more circumferentially oriented fiber directions in the microporous matrix restricted the radial expansion of the macroporous fibers, and, therefore, of the entire composite graft. Using a material more elastic than PCL, such as poly(l‐lactide‐co‐caprolactone) (PLCL), could potentially avoid this problem if the resulting microporous layer is more compliant than the macroporous one. The same holds for a microporous layer eventually obtained with another fabrication technique, e.g., by electrospinning. Gelatine provides effective cell adhesion sites,^[^ ^60^ ^]^ is considered to support the formation of the endothelium^[^ ^58^ ^]^ and shows promising hemocompatibility.^[^ ^61^ ^]^ Indeed, platelet adhesion was reduced on gelatine versus ePTFE and fibrin (Figure 2G; Figure S4, Supporting Information). We found hemolysis to be in the range of the reference materials (Figure 2H). Coagulation time of the gelatine matrix was slightly shorter than that of ePTFE but similar to the negative control (Figure 2I). Hemocompatibility could further be improved by functionalizing the gelatine matrix with anticoagulants such as heparin.^[^ ^62^, ^63^ ^]^ Successful formation of an endothelial cell layer lining the lumen and alignment of the cells in the direction of flow was found after 5 days of dynamic culture of the tubular constructs in a custom‐made flow setup (Figure 2J). The effect of combined cyclic stretching and shear stress resulting from a compliant graft under pulsatile flow has been investigated by Toda et al., who showed that endothelial cells remained viable and confluent after 24 h of dynamic culture, exhibiting elongation and alignment parallel to the flow and perpendicular to the stretch direction.^[^ ^64^ ^]^ The immune response of gelatine and PCL was analysed in vitro by cytokine release of pro‐inflammatory IL‐6 and anti‐inflammatory TGF‐β (Figure S5A, B, Supporting Information), as well as immunostaining of the surface markers CD206 and CD86 (Figure S5C, Supporting Information). Fibrin and ePTFE were used as reference materials. The cytokine release of TGF‐β was not significantly different for any of the tested materials. The cytokine release of IL‐6 was slightly higher for gelatine compared to the PCL and the reference materials; however, still significantly lower than the M1 control, indicating an overall low inflammatory response. Immunostaining confirmed expression of both the anti‐inflammatory marker CD206 and the pro‐inflammatory marker CD86 for all tested materials, with the CD86 expression being lower than that observed in the M1 control.

Upon implantation, the grafts need to withstand the dynamic physiological pressure levels. Burst pressure was determined to be 1411 ± 280 mmHg, which is in the range of the human saphenous vein (1599 ± 877 mmHg),^[^ ^46^ ^]^ but lower than that of the human internal mammary artery (3196 ± 1264 mmHg)^[^ ^46^ ^]^ (Figure 2K). There is no minimal burst pressure defined by the ISO 7198.

Next, we quantified the suture retention force to be 0.7 ± 0.3 N (Figure 2L). Although this value is below those reported for the human saphenous vein (1.92 ± 0.02 N)^[^ ^46^ ^]^ and internal mammary artery (1.35 ± 0.49 N),^[^ ^46^ ^]^ suturing the grafts to a porcine right coronary artery (Figure 2M) by an experienced cardiac surgeon was unproblematic. The anastomosis to the coronary artery was performed in the end‐to‐side fashion as it would be done for a coronary artery bypass graft (CABG). Anastomosed grafts were pressurized under physiological dynamic pressures and did not show acute anastomotic instabilities. Clinical literature reports grafts with similar suture retention force and successful implantation in animals.^[^ ^14^, ^65^, ^66^ ^]^ Our tubular scaffolds could be handled conveniently (Video S1, Supporting Information) and could be cut to the required length at any graft position without losing total integrity as the individual PCL fibers fused at their crossing points. Furthermore, grafts were sutured to human saphenous veins (Figure 2N) and to porcine femoral arteries (Figure 2O) in an end‐to‐end style. In both cases, the grafts pulsated at the anastomoses with corresponding amplitude as the corresponding native vessels when subjected to pulsatile flow.

In contrast, Federici et al. had to use a stiff connection device to anastomose their graft (fixed MEW fiber pattern covered by an electrospun layer) to a rat abdominal aorta as securing the graft via the eversion method had failed.^[^ ^39^ ^]^ While we used a running suture, it is evident that the suture style affects anastomotic compliance.^[^ ^67^ ^]^ In this context, it could be beneficial to apply degradable suture materials or suture‐less approaches, such as glueing, to avoid jeopardizing the anastomotic compliance.^[^ ^68^, ^69^ ^]^

It is crucial to prevent kinking of vascular prostheses, for example, when implanted in a loop configuration for vascular access or as an interposition graft in a vessel along a bending joint. ISO 7198 suggests a reduction of 50% of the luminal diameter as the threshold for kinking. Here, we found no reductions of the grafts’ outer diameter larger than 50% even for bending radii as low as 2.5 mm (Figure 2P). Under the assumption of an incompressible wall thickness, kinking, therefore, did not occur. This excellent anti‐kinking behavior inherently originates from the intertwined multi‐helix design of the microfiber MEW architecture. Similarly, although at a much larger scale, Wu et al. identified a single helical pattern to be the optimum anti‐kinking reinforcement pattern, which was printed onto electrospun grafts via fused filament fabrication.^[^ ^70^ ^]^ However, the effect of such reinforcement on compliance was not evaluated by Wu et al. Helical reinforcements are commonly used as a measure to avoid kinking in medical devices, including ureteral stents, percutaneous drainage and ventilation tubes, and catheters.^[^ ^71^, ^72^, ^73^ ^]^ The helix configuration confers resistance to kinking and, if internally positioned, to collapse, while allowing flexibility, for example, to navigate sharp‐angled bends.

Although here demonstrated with gelatine for tissue engineering application, the MEW matrix could be embedded in a non‐degradable polymer, e.g., silicones or thermoplastic polyurethanes, to achieve a “classical” prosthesis as an alternative to the clinically available ones.

### 2.3 Fiber Architectures for Compliance Gradient in Arteriovenous Grafts

Patients with end‐stage renal disease rely on functional vascular access (VA) to receive successful hemodialysis. As first option for VA, international guidelines propose the creation of an autogenous arteriovenous fistula (AVF).^[^ ^74^, ^75^ ^]^ However, AVFs are plagued inter alia with long maturation times of several months^[^ ^76^ ^]^ and high primary failure rates, particularly in the elderly.^[^ ^77^ ^]^ Consequently, and in light of a more patient‐centered approach to VA, the “fistula first” strategy has been challenged.^[^ ^78^, ^79^, ^80^ ^]^

**Figure 3:**
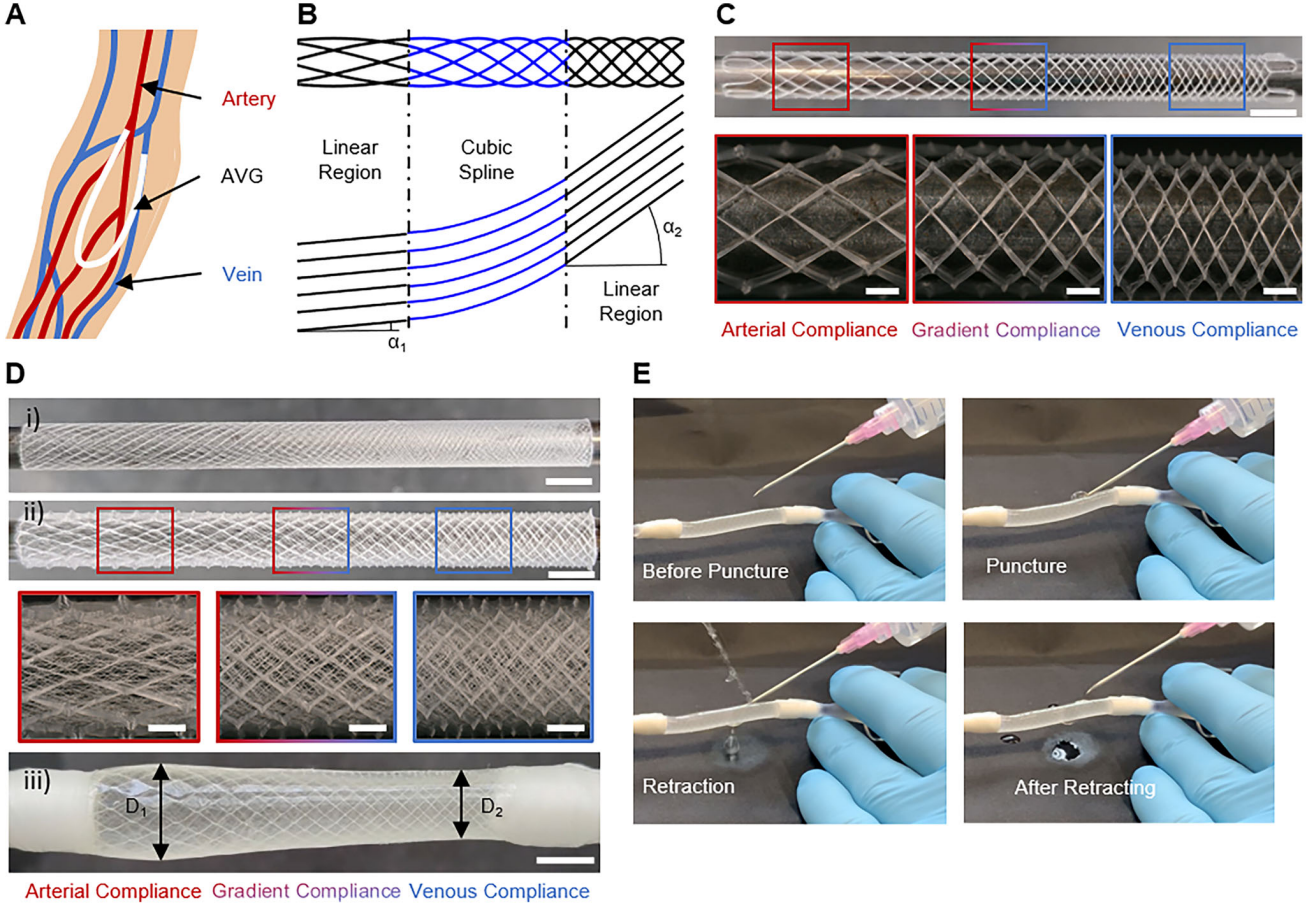
Gradient‐compliance scaffolds for arteriovenous grafts. A) AVGs connect an artery and a vein, usually in a loop configuration in the forearm. B) To accommodate the two distinct compliances (artery vs. vein) at the ends, the grafts feature a central gradient region. The schematic demonstrates the connection of the terminal linear fiber patterns (α_1_ vs. α_2_) by a cubic spline to result in a continuous fiber path for MEW. C) Melt‐electrowritten macroporous gradient scaffolds (fiber angle gradient from 30° to 60°) with magnified images highlighting the different fiber patterns within the same graft (scale bars 4 and 1 mm). D) The gradient fiber architecture was translated to the microporous matrix as well (i) to obtain a composite graft (ii). The magnified images show the fiber angle gradient of the macroporous layer (from 15° to 45°) and the corresponding gradient in the microporous component. The resulting gradient compliance (iii) is manifested by diameters varying along the axial direction despite a constant luminal pressure (scale bars 4 and 1 mm). E) The grafts were successfully punctured with an 18 G needle and leak‐free after cannulation.

Alternatively, VA can be accomplished via arteriovenous grafts (AVGs), where a prosthetic conduit is surgically interpositioned between an artery and a vein, two distinctly different vessels in terms of compliance, and usually placed in the patient's non‐dominant forearm (**Figure** **3**A). Using synthetic AVGs circumvents the problematic maturation time and performs well in the short term.^[^ ^9^ ^]^ However, synthetic AVGs are prone to develop neointimal hyperplasia, typically at the vein anastomosis, potentially leading to stenotic failure.^[^ ^9^, ^10^, ^11^, ^81^, ^82^ ^]^ Therefore, tissue‐engineering such grafts has been identified as one of today's clinical needs.^[^ ^83^ ^]^

Here, we introduce a spatially heterogeneous fiber architecture to obtain an AVG with a compliance gradient to mediate between venous and arterial compliance while matching the compliance at both the anastomosis sites. This graft design consists of three regions: i) arterial compliance, ii) venous compliance, and iii) a gradient region to provide a mechanically smooth transition between regions i) and ii). The gradient region was obtained by designing a fiber path following a cubic spline that tangentially connects the linear fiber segments at the graft ends, where either a venous or arterial compliance prevails (Figure 3B,C). More information on the algorithm is provided in the supplementary information (Figures S7–S9, Supporting Information). This gradient approach was translated to both the microporous and the macroporous fiber architecture (Figure 3D). To maintain the graft's diameter constant along the length, in the region with the larger fiber angle (venous region with lower compliance), the distance between parallel fibers will be decreased. This geometric restriction is beneficial for the compliance gradient, since the slight difference in fiber density only further supports the compliance difference between venous and arterial regions.

A luminal pressure of 120 mmHg resulted in different graft diameters at the arterial and venous sides with a smooth transition in diameter along the graft's central region (Figure 3D), reflecting the compliance gradient.

Furthermore, the grafts were successfully cannulated using an 18 G needle while being pressurized to 120 mmHg. After retracting the needle, the graft was self‐sealing (Figure 3E; Video S2, Supporting Information). Once implanted, this will be further supported by hemostatic effects and tissue healing.

For the first time, this work presents a gradient tubular scaffold architecture via MEW to enable a smooth transition of mechanical properties between two distinctly different regions. So far, MEW scaffolds with spatially heterogeneous patterns have been shown in‐plane^[^ ^21^, ^84^, ^85^ ^]^ and across the scaffolds’ thickness direction.^[^ ^37^, ^86^, ^87^, ^88^, ^89^ ^]^ However, they were strictly separated in regions with discrete mechanical properties, and a transitional region was missing. Although demonstrated for flat scaffolds in the context of MEW scaffolds for heart valves, Vernon et al. advocated for the importance of engineering interfaces between regions as these are potential regions for the onset of mechanical failure and pointed to the superior performance of continuous fiber interfaces as compared to overlapping or suture‐like interfaces.^[^ ^22^ ^]^ Here, we avoid the problem of interfaces by a smooth gradient transitional pattern between regions of different mechanical characteristics.

Although we have extensively demonstrated the structure‐property relationship of our design concept, the evaluation of the effects of compliance on the formation of tissue and vice versa in vivo is the next needed step.

## 3 Conclusion

This work introduces compliant small‐diameter vascular grafts by design, based on an intertwined multi‐helix arrangement of polymeric microfibers fabricated via MEW. Leveraging the tunable structure‐property relationship of MEW fiber architectures, we control the fiber winding angle to realize grafts with compliance matching the range reported for human vessels from veins to arteries. This holds the potential of avoiding a compliance mismatch, which has been identified as a major driver for implant failure. Building on that approach, we introduced a gradient architecture to obtain tailored grafts with gradually transitioning compliance from arterial to venous values along their length. This could be of particular benefit for in situ tissue‐engineered arteriovenous grafts. By combining excellent handling and anti‐kinking behavior with adequate suture retention force, burst strength, and hemocompatibility, this technology platform provides promising aspects for clinical translation.

## 4 Experimental Section

### G‐Code Generator

Our previously reported software platform, based on MATLAB R2020b (The Mathworks, USA), outputs G‐code commands tailored to MEW of super‐positioned, angularly shifted sets of parallel fiber lines.^[^ ^35^ ^]^ Here, this platform was upgraded by combining two regions of different linear fiber architectures via the integration of cubic splines to result in a gradient spatially heterogeneous fiber pattern. A detailed description is provided in the supporting information.

### Melt Electrowriting

MEW was performed on an in‐house developed setup. Briefly, the system consisted of two linear mechanical‐bearing screw‐driven stages (PRO115SL, Aerotech, Germany) for the *X* and *Z* direction and a direct‐drive rotary stage (ADRS100, Aerotech, Germany) for rotation in the Y direction (circumferential direction of the grafts) that were controlled by Automation1 Studio (Aerotech, Germany). The MEW print head was mounted on the *Z*‐axis and was designed to host a syringe (3cc Optimum, Nordson EFD, USA) equipped with a 23 G needle (Nordson EFD, USA) protruding ≈1 mm from the print head. Both the syringe and the needle were individually heated via a temperature control unit (MIUE04003F/MIUE06012D and MI8844820G, Hotset, Germany). Polymer extrusion was driven by pressurized air applied to the syringe via an electro‐pneumatic controller (ITV1050‐31F1CL3, SMC, Japan), and fiber formation was initiated by applying a positive electrical potential (MPL 200‐30000 POS, FuG Elektronik, Germany) to the needle while the cylindrical metal collector (4 mm diameter) was electrically grounded.

Medical grade poly(ɛ‐caprolactone) (Purasorb PC12, Lot# 2004002576, Corbion, Netherlands) was melted at 75 °C in the syringe and extruded via the needle at 85 °C. MEW was performed at a working distance of 4.0 mm between the print head and the collector with a voltage of 5.6 kV, print speed of 210 mm min^−1^, and air pressure of 2 bar for the macroporous patterns and 5.3 kV, 440 mm min^−1^, and 0.5 bar for the microporous patterns.

### Composite Grafts

The multiscale scaffolds were composed of a melt‐electrowritten microporous fiber matrix and a macroporous fiber architecture, both embedded in gelatine. The microporous pattern had five stacks of parallel fiber sets (200 µm inter fiber distance) shifted ±5° and ±15° relative to the main fiber winding angle of the macroporous fiber pattern (e.g. ±[30°, 40°, 50°, 60°] for a ±45° macroporous pattern). The macroporous pattern consisted of 15 layers of intertwined helices with 750 ± 50 µm interfiber distance. For dip‐coating, 5 g type A gelatine from porcine skin (Sigma Aldrich, USA) was dissolved in 50 mL of deionized water under constant stirring (60 min, 400 rpm) at 60 °C. Separately, 350 µg transglutaminase (BDF Natural Ingredients, Spain) was dissolved in 1 mL of deionized water. The transglutaminase solution was added to the gelatine solution at a ratio of 1:100. Heating was stopped and once the gelatine solution reached 35 °C, the MEW scaffolds, still sitting on the collector, were dip‐coated. To ensure consistent gelatine thickness, the scaffolds were kept rotating while cooling down to room temperature.

### Compliance

Compliance (*n* = 3, *i* = 4) was measured in accordance with ISO 7198. Here, the scaffolds were mounted into a measurement chamber orthogonally to the light path of an optical micrometer (LS‐7030(M), Keyence, Germany). The scaffolds were closed on one outlet side of the measurement chamber, while via the other side, pressure was applied using a programmable syringe pump (LA120, Harvard Apparatus, USA) and monitored via a pressure sensor (Xtrans, Codan Medizinische Geräte, Germany). For the macroporous scaffolds only, a silicone liner was inserted into the lumen in agreement with ISO 7198. The compliance of the silicone liner was 25.7 ± 3.5%(100 mmHg)^−1^.

While applying a pulsatile pressure profile (80 to 120 mmHg, 60 ± 10 pulses per minute) to the graft, changes in its diameter were recorded by the optical micrometer. A Python script collected all data, and a MATLAB script calculated the dynamic compliance according to ISO 7198:

(1) $$C=\frac{\left(r_{p_{2}}-r_{p_{1}}\right)/r_{p_{1}}}{\left(p_{2}-p_{1}\right)}\times 10^{4}$$

where *p*_1_ is the lower internal pressure and $r_{p_{1}}$ the corresponding radius and *p*_2_ is the higher internal pressure and $r_{p_{2}}$ the corresponding radius.

### Suture Retention

Suture retention was determined following ISO 7198 by inserting a 7‐0 polypropylene suture (Covidien Surgipro II VP‐704‐MX, Medtronic, Ireland) ≈2 mm from the graft's end and mounting the suture to the moving clamp of a tensile testing machine (Zwickline Z2.5, 10 N load cell, ZwickRoell, Germany). The opposite side of the graft was immobilized in the other clamp of the tensile testing machine. The measurement was performed at a cross‐head speed of 50 mm min^−1^. Composite grafts with 45° (*n* = 4) and 15° (*n* = 3) fiber patterns were tested. Grafts were also sutured to human saphenous veins, porcine right coronary arteries, and porcine femoral arteries via a running suture (8‐0 polypropylene, Prolene, Ethicon, USA).

### Burst Pressure

Burst pressure (*n* = 5) was assessed according to ISO 7198. A planar sample (1 cm × 1 cm) was excised from composite grafts with a 45° fiber pattern and placed inside a testing chamber, featuring a 4 mm diameter hole against which the samples were pressed by a pressure increasing at a rate of 83 ± 5 mmHg s^−1^ applied via a syringe. Pressure was recorded via a pressure sensor (MIDAS 401 001/000, Jumo, Germany), and the peak pressure at failure was recorded as the burst pressure.

### Kinking Radius

To evaluate the kinking behavior, composite grafts (*n* = 4, ± 30° main fiber winding angle) were pressurized with 100 mmHg and bent around a template featuring radii from 2.5 to 15 mm. The reduction in diameter was quantified via:

(2) $$\Delta D=100\%\;\times \frac{\left(D_{0}-D_{1}\right)}{D_{0}}$$

where *D*_0_ is the graft's diameter in relaxed configuration and *D*_1_ is the graft's diameter while undergoing bending.

### Cannulation

Grafts were pressurized with 120 mmHg and punctured with an 18 G needle (L1 0103, Servoprax, Germany) at a 45° angle with respect to the longitudinal graft axis and the needle cut facing upwards.

### Hemolysis

Citrated whole blood, 200 µL was diluted in 10 mL of 0.9% saline solution (B. Braun, Germany). The samples (*n* = 5, ≈ 210 mm^2^ surface area, high‐density PE (Raumedic, Germany) and ePTFE (GORE‐TEX Cardiovascular Patch, W. L. Gore & Associates, USA) as reference materials) were incubated in 700 µL diluted blood under gentle agitation at 37 °C for 2 h. Whole blood incubated with distilled water or saline solution served as the positive control (PC) and negative control (NC), respectively. After centrifugation, the absorbance values of the supernatants were measured at 545 nm with a spectrophotometer (Spark, Tecan, Switzerland). Hemolysis rate was calculated using the following formula according to ASTM F 756:^[^ ^90^ ^]^

(3) $$\begin{array}{c} \%\;\mathrm{Hemolysis}\;=\frac{\mathrm{Absorbance}\;\mathrm{of}\;\mathrm{test}\;\mathrm{sample}-\mathrm{Absorbance}\;\mathrm{of}\;\mathrm{NC}}{\mathrm{Absorbance}\;\mathrm{of}\;\mathrm{PC}-\mathrm{Absorbance}\;\mathrm{of}\;\mathrm{NC}}\times 100 \end{array}$$

### Coagulation Time

To assess the plasma recalcification time, the samples (*n* = 5, discs with 12 mm diameter) were incubated with 250 µL platelet‐poor plasma (PPP) derived from citrated whole blood at 37 °C for 20 min. Fibrin (prepared by mixing fibrinogen and thrombin as reported previously)^[^ ^91^ ^]^ served as PC, while PPP was used as NC, and ePTFE (GORE‐TEX Cardiovascular Patch, W. L. Gore & Associates, USA) as reference. Upon addition of 100 µL CaCl2 to 100 µL of supernatant of each sample, the absorbance was measured with a spectrophotometer (Spark, Tecan, Switzerland) at 405 nm in 20 s intervals for 2 h to assess the time required to obtain a 5% increase in fibrin formation as compared to the total increase.

### Platelet Adhesion

Platelet‐rich plasma (PRP), 600 µL, was added to the samples (*n* = 3, fibrin^[^ ^91^ ^]^ and ePTFE (GORE‐TEX Cardiovascular Patch, W. L. Gore & Associates, USA) as references) and incubated at 37 °C for 1 h. The samples were washed in PBS and fixated with 2% glutaraldehyde prepared in PHEM buffer. They were then dehydrated by a series of increasing concentrations of ethanol (30%, 50%, 70%, and 3 × 100%) each for 15 min at room temperature and subsequent critical point drying (BAL‐TEC GmbH, Germany). The samples were sputter‐coated (7 nm gold layer) and imaged using scanning electron microscopy (accelerating voltage 10 kV, JSM‐6390, Jeol, Germany), and the surface area covered by platelets was assessed using Fiji^[^ ^92^ ^]^ plugins in ImageJ.^[^ ^93^ ^]^ For each of the three independent samples, three image segments of an approximate size of 43 µm × 32 µm were evaluated.

### Endothelialization

For the dynamic endothelialization assessment, human umbilical vein endothelial cells (HUVEC) were used. The cells were cultured in endothelial cell basal medium (EBM‐2, Lonza Group AG, Basel, Switzerland) supplemented with the endothelial growth medium 2 kit. Supplements included 0.1% ascorbic acid, 0.4% human fibroblast growth factor, 0.1% insulin, 0.1% gentamicin, 2% fetal bovine serum (FBS), 0.1% endothelial growth factor, 0.04% hydrocortisone, 0.1% epidermal growth factor, and 0.1% heparin. The polymer scaffold was washed for 30 min using 70% ethanol and 30 min using sterile phosphate‐buffered saline solution (PBS). It was then dip‐coated in sterilely prepared gelatine, and after complete crosslinking, it was positioned in sterile silicone tubing. The cells were seeded on the lumen of the tubular graft at a density of 250 000 cells cm^−2^. The graft was clamped shut on both ends and incubated at 37 °C for 6 h. Every 30 min, the graft was rotated 90° around its axis to ensure even distribution of the cells on the lumen. The graft was then transferred into a custom‐made flow setup, which enables precise control of flow and pressure conditions. Over 2 days, the flow was gradually increased up to 250 mL min^−1^, corresponding to a shear stress of 5.3 dynes cm^−2^, and a pulsatile pressure profile of 80 to 120 mmHg. The graft was kept under these flow conditions for another three days. The graft was then removed from the silicone tubing and prepared for immunostaining. The sample was fixed in 4% paraformaldehyde (Carl Roth, Germany) at room temperature for 15 min and washed in PBS three times each for 5 min. Blocking was performed in 5% normal goat serum solution (DAKO GmbH, Jena, Germany) for 1 h at room temperature. The graft was incubated with the mouse anti‐human primary antibody CD31 (Sigma, Germany) for 1 h at room temperature. After three washes in PBS for 5 min each, the graft was incubated in Alexa A594 secondary antibody for 1 h at room temperature. The sample was washed three times for 5 min in PBS and counterstained with DAPI nuclei acid stain (Carl Roth, Germany). The graft was carefully cut open along the longitudinal axis, and images were taken with a fluorescence microscope BZ‐X800 (Keyence Corporation, Japan).

### Microscopy and Image Analysis

Scaffolds were imaged using a digital light microscope (VHX‐5000, Keyence, Germany), and fiber diameter (*n* = 4, *i* = 3) was determined via the built‐in software of the microscope. Pore sizes were evaluated using light microscopy images. An in‐house developed MATLAB script segmented the pores from light microscopy images (*n* = 3, *i* = 100). Pixels per pore were counted and scaled to derive an area per pore. Under the assumption of a circular pore morphology, a pore radius was calculated from the area per pore.

### Ethical Approval

Leftover anonymized human vein segments were obtained from CABG surgery performed at the Department of Cardiac Surgery at Ludwig‐Maximilian University, Munich. Tissue was solely used if the patient's informed consent was given beforehand. This procedure was approved by the ethics committee of Ludwig‐Maximilian University Munich (259‐15). Porcine tissue was sourced from a local abattoir. Human blood was collected from informed donors following approval by the ethics committee of the Technical University of Munich (2023‐40‐S‐SR). Human umbilical vein endothelial cells were collected and used in accordance with approval by the ethics committee of the Technical University of Munich (2023‐531‐S‐KH).

### Statistics

Statistical analysis was performed with Prism 9.2.0 (GraphPad Software, USA). After confirmation of normal distribution ANOVA, with post hoc Tukey multiple comparisons, was performed. Values are reported as mean ± standard deviation. In Figure 2K,L, literature values for human vessels were used, and a normal distribution was assumed for this data to perform statistical analysis. ****: *p* ≤ 0.0001, ***: 0.0001 < *p* ≤ 0.001, **: 0.001 < *p* ≤ 0.01, and *: 0.01 < *p* ≤ 0.05 were used to indicate the level of significance.

## Conflict of Interest

The authors declare no conflict of interest.

## Supporting information



Supporting Information

Supplementary Video 1

Supplementary Video 2

## Data Availability

The data that support the findings of this study are available from the corresponding author upon reasonable request.
