# Supplementary material for: Programmable Compliance in Small‐Diameter Vascular Grafts by Design of Melt‐Electrowritten Scaffold Architectures for In Situ Tissue Engineering
Source: Adv Healthc Mater. 2025 Sep 12;15(2):e02038. doi: 10.1002/adhm.202502038 (PMC12805616; doi:10.1002/adhm.202502038)
Supplement: Supplementary file 1 — Supporting Information [file ADHM-15-0-s002.pdf]

ADVANCED HEALTHCARE MATERIALS

Supporting Information

for *Adv. Healthcare Mater.*, DOI 10.1002/adhm.202502038

Programmable Compliance in Small-Diameter Vascular Grafts by Design of Melt-Electrowritten Scaffold Architectures for In Situ Tissue Engineering

*Kilian Maria Arthur Mueller, Christina Ahrens, Linda Grefen, Salma Mansi, Dario Arcuti, Elena De-Juan-Pardo, Felix Kur, Christian Hagl and Petra Mela**

Supporting Information

Programmable Compliance in Small-diameter Vascular Grafts by Design of Melt-electrowritten Scaffold Architectures for *In Situ* Tissue Engineering

Kilian Maria Arthur Mueller [a,+]

Christina Ahrens [a,+]

Linda Grefen [b, c]

Salma Mansi [a]

Dario Arcuti [a]

Elena De-Juan-Pardo [d,e,f]

Felix Kur [b]

Christian Hagl [b]

Petra Mela [a*]

[a] Technical University of Munich, Germany; TUM School of Engineering and Design, Department of Mechanical Engineering, Chair of Medical Materials and Implants; Munich Institute of Biomedical Engineering (MIBE); Munich Institute of Integrated Materials, Energy and Process Engineering (MEP)

[b] Department of Cardiac Surgery, LMU University Hospital, Munich, Germany

[c] DZHK (German Centre for Cardiovascular Research), partner site Munich Heart Alliance, Munich, Germany

[d] T3mPLATE, Harry Perkins Institute of Medical Research, Queen Elizabeth II Medical Centre and University of Western Australia Centre for Medical Research, The University of Western Australia, Perth, WA 6009, Australia

[e] School of Engineering, The University of Western Australia, Perth, WA 6009, Australia

[f] Curtin Medical School, Curtin University, Perth, WA 6102, Australia

[+] Equal contribution

* Corresponding author

Compliance for Different Pressure Ranges

Method:

Compliance of the microporous scaffolds with winding angles from 15° to 75° was measured for different pressure profiles in the custom-made pressure setup described in the manuscript. As suggested by ISO 7198, the grafts were subjected to different pulsatile pressure profiles, specifically: 50 to 90 mmHg, 80 to 120 mmHg, and 110 to 150 mmHg. The compliance was determined for each profile as described in the Materials and Methods section of the manuscript.

Results:

Testing the grafts at different pressure profiles showed a slight trend towards increasing compliance for scaffolds with low fiber winding angles (15° and 22.5°), while higher fiber angles (30°, 45°, 60°, 75°) presented a minor trend towards decreasing compliance for an increase in mean pressure.

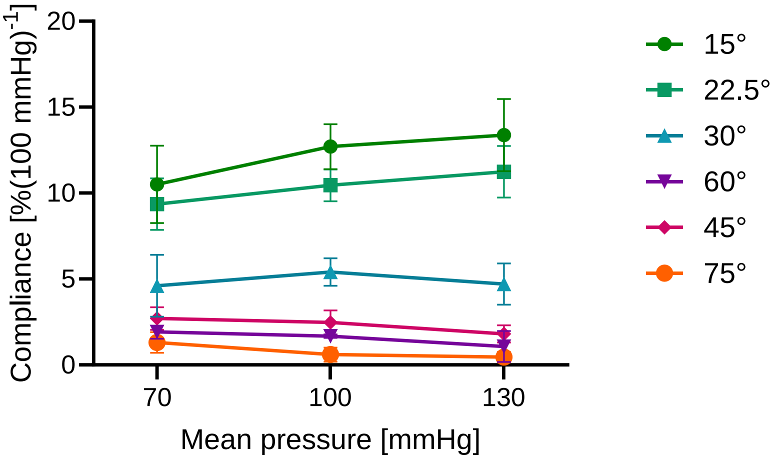

Figure S1: Compliance of macroporous scaffolds with winding angles from 15° to 75° when subjected to different pressure profiles (i.e: 50 to 90 mmHg, 80 to 120 mmHg and 110 to 150 mmHg) as suggested by ISO 7198. The mean pressure of each interval is indicated.

Fatigue Testing

Method:

Fatigue behaviour was tested on three grafts with distinct compliances (15°, 30° and 60° winding angle). The grafts were mounted in the custom-made compliance measurement setup as described in the manuscript. A cyclic pressure profile of 80 to 120 mmHg at 2Hz was applied and the grafts were tested for seven consecutive days. Compliance measurements were taken (as described in the manuscript) during this time to monitor the fatigue behaviour.

Results:

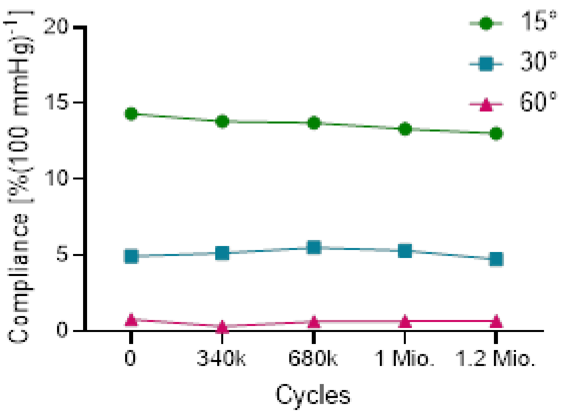

Figure S2: Compliance of three composite grafts with 15°, 30° and 60° winding angles after cyclic testing for 1.2 million cycles. The grafts did not show changes in compliance that would indicate structural fatigue.

Isotropic Microporous Matrix

Method:

To analyze the influence of an isotropic microporous matrix in contrast to the one matching the main fiber direction of the macroporous layer, composite grafts were fabricated featuring an isotropic matrix layer with fiber winding angles ± [11.25°, 33.75°, 56.25°, 78.75°]. This experiment was exemplary performed for grafts with winding angles of 15° and 30° (n=3) of the macroporous layer.

Results:

The isotropic microporous layer restricted the compliance significantly with respect to the microporous layer matching the winding angle of the macroporous layer.

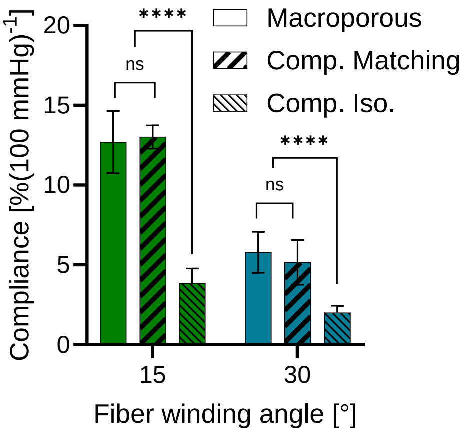

Figure S3: Compliance measurement for macroporous scaffolds and composite grafts featuring a compliance matching microporous layer (Comp. Matching) or an isotropic microporous layer (Comp. Iso.).

Platelet Adhesion

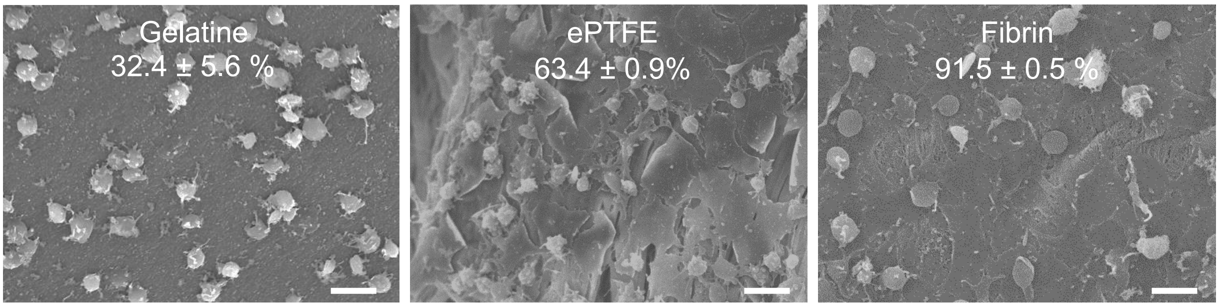

Figure S4: SEM images of platelet adhesion on gelatine, ePTFE and fibrin (scale bar: 5 µm).

Inflammatory Response Analysis

Method:

Inflammatory response was evaluated by cytokine release and immunostaining of CD86 and CD206 surface markers. Human cell line THP-1 (ATCC, Germany) from peripheral blood of an acute monocytic leukemia patient were cultured in Roswell Park Memorial Institute (RPMI) 1640 medium (Gibco, Life Technologies, UK) supplemented with 10% v/v fetal calf serum (Gibco, Life Technologies, UK), l-glutamine (2 mM, Gibco, Life Technologies, UK), sodium pyruvate (1 mM, Gibco, Life Technologies, USA), penicillin (1 U mL^{-1} , Gibco, Life Technologies, USA), and streptomycin (1 µg mL^{-1} , Gibco, Life Technologies, USA). Cells were differentiated into M0 macrophages by incubating in complete culture medium supplemented with phorbol 12-myristate 13-acetate (100 nm, Sigma-Aldrich) for 72 hours. After differentiation, M0 macrophages were rested in complete culture medium for 24 hours, and then

seeded at a density of 450 000 cells cm^{-2} on the samples. THP-1 macrophages were polarized for 48 hours in complete culture medium supplemented with 1 ug ml-1 IFNg and 1 ug ml-1 LPS (Sigma Aldrich, Germany) to M1 macrophages, or with 20 ng ml-1 IL-4 and 20 ng ml-1 IL-13 (Gibco, Fisher Scientific GmbH, Germany) to M2 macrophages, respectively, and served as controls. The cytokine concentration in the cell culture supernatant was determined on day 3. Released proinflammatory (IL-6) and anti-inflammatory (TGF-β) cytokines were quantified with the DuoSet enzyme-linked immunosorbent assay (ELISA) kits (R&D Systems, Minneapolis, MN,USA) following the manufacturer's instructions. The absorbance was measured using a Multimode Microplate Reader (Tecan, Spark) at 450 nm. For immunostaining, the samples were rinsed three times 5 minutes in phosphate buffered saline (PBS) and fixed in 4 % formaldehyde (Carl Roth, Germany) in PBS. Then, after rinsing three times 5 minutes in PBS, the samples were blocked in 5 % normal goat serum (Dako, Germany) in PBS for 1 hour at room temperature. After removing the blocking solution, the samples were incubated in the primary antibody solutions containing mouse anti-human CD86 (10 µg ml^{-1}, abcam, Cambridge, UK) and rabbit anti-human CD206 (1 µg ml^{-1}, abcam, Cambridge, UK) for 1 hour at room temperature. After rinsing three times 5 minutes in PBS, the samples were incubated in the secondary antibody solutions containing goat anti-mouse Alexa Fluor 594 (5 µg ml^{-1}, Invitrogen, ThermoFisher Scientific, Life Technologies, Eugene, USA) and goat anti-rabbit Alexa Fluor 488 (5 µg ml^{-1}, Invitrogen, ThermoFisher Scientific, Life Technologies, Eugene, USA) for 1 hour at room temperature. After rinsing three times 5 minutes in PBS, nuclei staining was conducted by incubating in a 0.2 µg ml^{-1} DAPI (Carl Roth, Germany) in PBS solution for 5 minutes at room temperature. Finally, the samples were rinsed three times 5 minutes in PBS and then imaged under the fluorescence microscope BZ-X800 (Keyence, Neu-Isenburg, Germany).

Results:

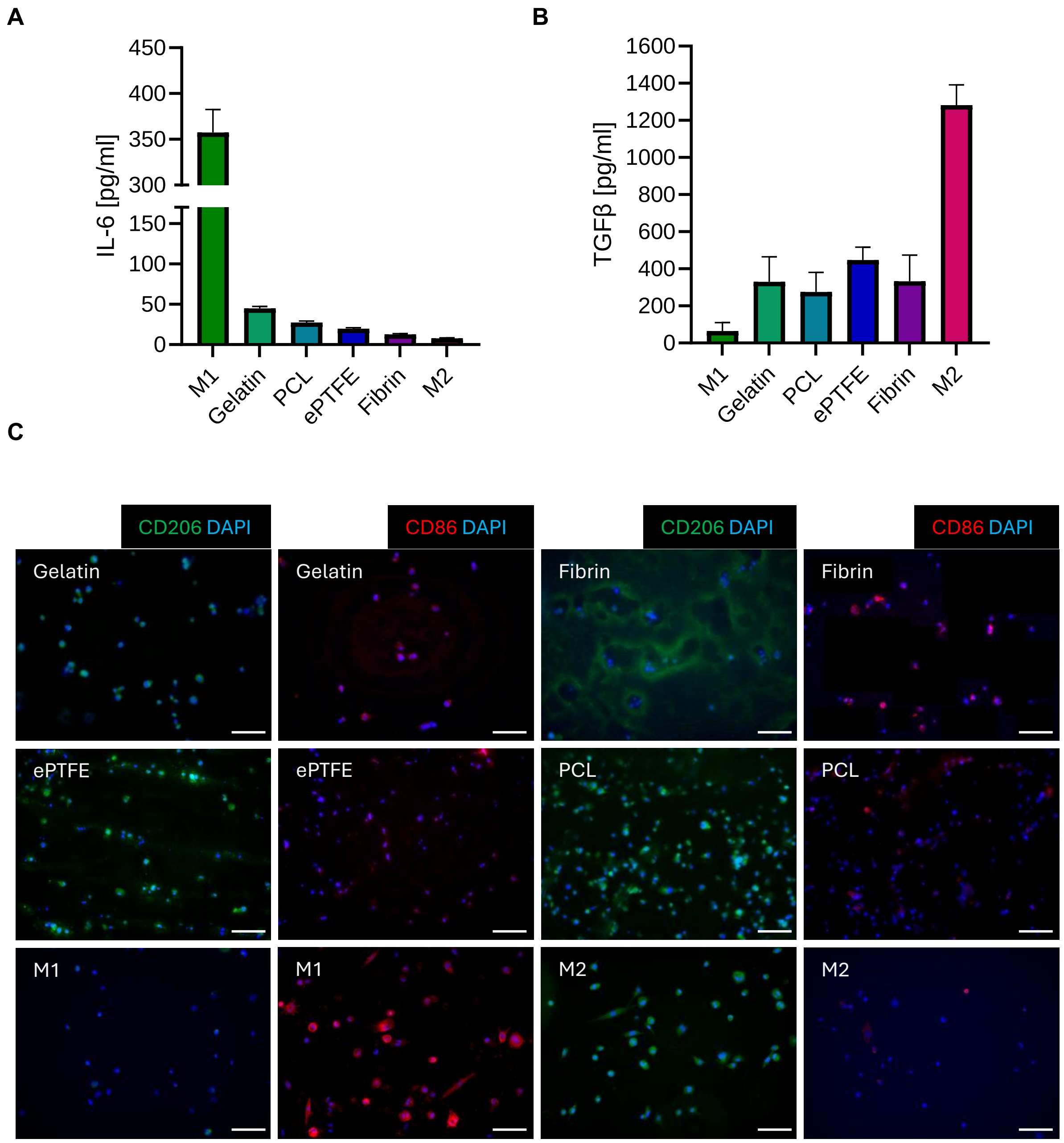

Figure S5: Inflammatory response analysis of gelatine, PCL, ePTFE and fibrin. Macrophages polarized to M1 and M2 in wells function as controls. Cytokine concentration of A) IL-6 and B) TGFβ determined by ELISA. C) Immunostaining of CD206 and CD86. DAPI was used for staining of nuclei (scale bar: 100 µm).

Description of the Algorithm for Gradient Grafts

The algorithm builds a line-based pattern depending on input parameters, including winding angles, fiber distance, and layer height. With these parameters, Gcodes for tubular scaffolds consisting of three areas are generated:

- Region 1: pattern with constant fiber winding angle, resulting in a scaffold region with constant compliance.
- Gradient Region: transition between region 1 and region 3 with changing fiber winding angle resulting in a compliance gradient.
- Region 3: pattern with constant fiber winding angle, resulting in a scaffold region with constant compliance different to the compliance in region 1

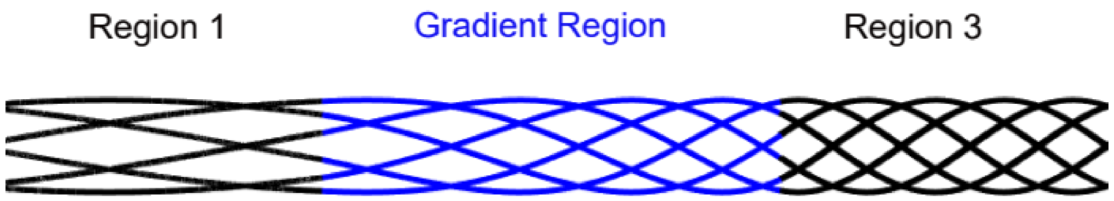

Figure S6: Tubular scaffold with double helix pattern consisting of three regions with distinct geometrical properties; two terminal regions with constant fiber winding angles are connected by a gradient region with a transitioning fiber winding angle.

1. **Generation of the Linear Pattern**

The fiber pattern for the two regions with constant fiber winding angles are generated based on the algorithm previously described by Mueller et al.[1] Here, sets of parallel linear fiber paths with user-defined fiber winding angle α form multiple intertwined helices. The parallel fiber paths are spaced equidistantly along the circumference of the tubular graft.

2. **Cubic Spline Interpolation**

Cubic splines connect the two regions with constant fiber winding angles. As the number of parallel lines (helices) in the two regions with constant fiber winding angles depends on their respective fiber winding angle, the line pattern is adjusted to enable spline interpolation before the spline interpolant is calculated. To this end the line count is calculated as

$$line\ count = \frac{circumference}{fiber\ distance} \times \cos{(\alpha)}$$

Consequently, the line count in the region with the smaller pitch angle (α_1) is adjusted to match the line count of the second region with the higher fiber winding angle (α_2). Next, the set of lines with the higher fiber winding angle (α_2) is offset along the y direction so that a straight line between the two line sets would have an inclination angle of $(\alpha_1 + \alpha_2)/2$.

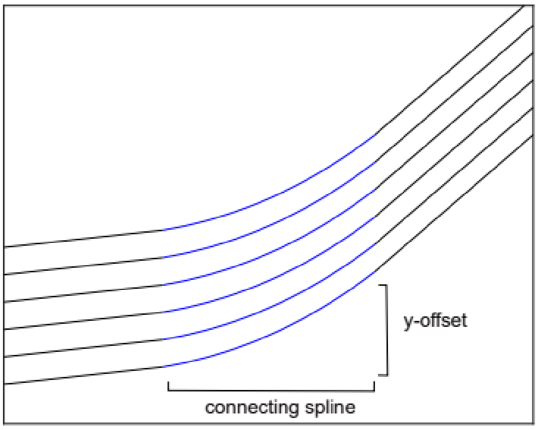

Figure S7: Line sets connected with a cubic spline in the gradient region of the scaffold. The spline leads to gradually changing pore morphologies in the grafts. For clarity, 3D tubular fiber pathways are projected into the flat XY plane.

The cubic spline is then calculated as an interpolation of three points on each line (starting point, center point, and end point), resulting in a cubic curve that aligns tangentially with the straight lines to generate a smooth connection. As the two-dimensional pattern will later be melt-electrowritten onto a three-dimensional cylindrical collector, the pattern has to be distributed evenly along the circumference of the cylindrical collector. Thereby, a geometrical constraint has to be taken into account: The distribution of lines in y-direction (circumferential direction of the cylindrical collector) cannot be bigger than the circumference of the cylindrical collector. The fiber spacing in the region with a higher fiber winding angle (α_2) is, therefore, adjusted accordingly.

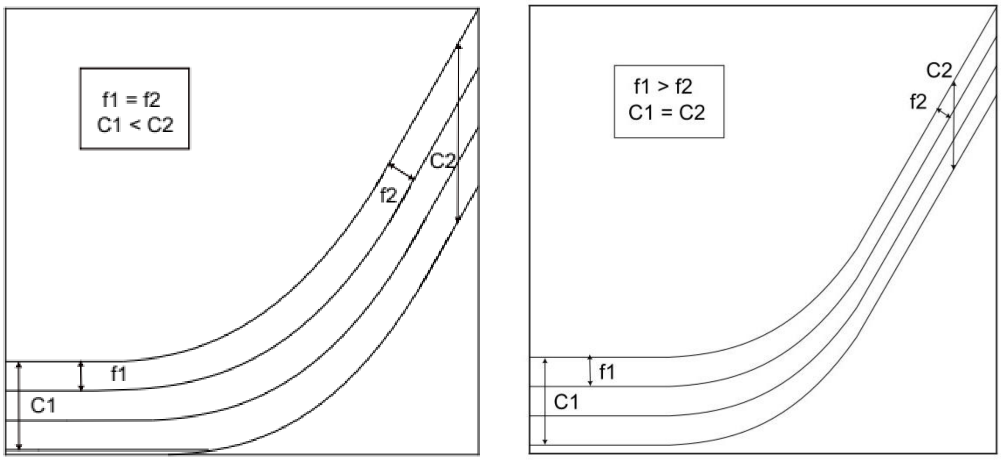

Figure S8: Adjustment of fiber spacing to maintain a constant circumferential dimension along the scaffold's longitudinal axis (f = fiber distance, C = circumference). Left) Initial set of lines with constant fiber distance ($f_1 = f_2$), resulting different circumferential dimensions ($C_1 < C_2$). Right) Adjusted fiber spacing ($f_1 > f_2$) to maintain a constant circumferential dimension ($C_1 = C_2$) along the scaffold's longitudinal axis. For clarity, 3D tubular fiber pathways are projected into the flat XY plane.

3. Connecting Curves

The individual line patterns are then connected via semicircles to generate a single continuous fiber path, as described in Mueller et al.[1]

4. Stacking Multiple Fiber Paths Results in Layered Scaffolds

Chosen fiber winding angles (α_1, α_2) can be mirror-matched by using their complementary negative angles ($-\alpha_1$, $-\alpha_2$). This results in diamond-shaped pores in between the fiber paths. Repeatedly generating fiber paths following the steps as described above and stacking them forms multilayered scaffolds.

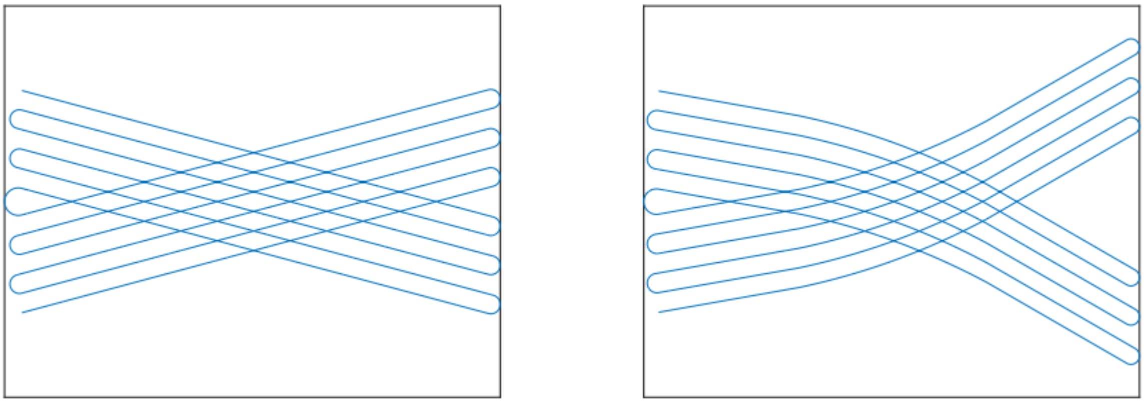

Figure 9: Left) Uniform 2D line pattern with +15° and -15° winding angles forming crosshatch-pores. Right) 2D line pattern with a gradient from ±10° to ±30° winding angles forming crosshatch-pores with changing morphology. For clarity, 3D tubular fiber pathways are projected into the flat XY plane.

References

[1] K. M. A. Mueller, A. Unterrainer, D. M. Rojas-González, E. De-Juan-Pardo, M. S. Willner, J. Herzen, P. Mela, *Adv. Mater. Technol.* **2023**, *8*, 2201158.
